# Knowledge of and Utilization of Emergency Contraceptive and Its Associated Factors among Women Seeking Induced Abortion in Public Hospitals, Eastern Tigray, Ethiopia, 2017: A Cross-Sectional Study

**DOI:** 10.1155/2019/7209274

**Published:** 2019-11-20

**Authors:** Desta Abraha, Guesh Welu, Meresa Berwo, Mulu Gebretsadik, Tesfay Tsegay, Gdiom Gebreheat, Hadush Gebremariam

**Affiliations:** ^1^Department of Midwifery, College of Medicine and Health Sciences, Adigrat University, Adigrat, Ethiopia; ^2^Department of Midwifery, College of Health Sciences and Comprehensive Specialized Hospital, Aksum University, Aksum, Ethiopia; ^3^Department of Nursing, College of Medicine and Health Sciences, Adigrat University, Adigrat, Ethiopia; ^4^School of Public Health, College of Health Sciences, Mekelle University, Mekelle, Ethiopia

## Abstract

**Background:**

In developing countries, most maternal deaths are related to the lack of accessibility and availability of reproductive health services. In those nations, emergency contraceptive pills are the most commonly used family planning methods to prevent unintended pregnancy. However, women do not use this family planning method for different reasons. Consequently, women expose to unsafe abortion which results in maternal morbidity and mortality.

**Objective:**

To assess the knowledge of and utilization of emergency contraceptive and its associated factors among women seeking induced abortion in public hospitals, Eastern Tigray, Ethiopia, 2017.

**Methods:**

Hospital-based cross-sectional study was conducted on 380 women, who came for safe termination of pregnancy from April to July 2017. Systematic random sampling technique was used. Pretested structured questionnaire was used to collect data through interview. Data were entered using Epi Info version 7 and exported to SPSS version 20 for analysis. Data were presented using descriptive statistics. Bivariate and multivariate logistic regression was carried out to see if there was significant association between variables at *P* < 0.05 and 95% confidence interval (CI).

**Result:**

Out of the total 369 respondents, 149 (40.4%) had the knowledge about emergency contraceptive pills. The magnitude of utilization of emergency contraceptive among respondents was found to be 45 (12.2%). Protestant in religion (AOR = 60.85, CI (5.34–693.29)), previous utilization of any contraceptive method (AOR = 0.13, CI (0.05–0.36)), and women who were not knowledgeable about emergency contraceptive (AOR = 0.030, CI (0.006–0.14)) were significantly associated with the utilization of emergency contraceptive.

**Conclusion:**

Most of the women were not knowledgeable about emergency contraceptive and utilization of emergency contraceptive was also very low. In conclusion, religion, knowledge, and previous utilization of emergency contraceptive were associated with the utilization of emergency contraceptive.

## 1. Background

Utilization of family planning is very useful to decrease unintended pregnancy and unsafe abortion which in turn reduces maternal morbidity and mortality [1]. Emergency contraceptive (EC) is indicated after unprotected sexual intercourse, following sexual abuse, misuse of regular contraception, or nonuse of contraception [2, 3]. It is prepared in different forms such as intrauterine devices (IUD) and emergency contraceptive pills (ECP) [4]. Worldwide, around 120 million women per year need contraception but they did not use it. Parallel with this, approximately 250 million pregnancies occur and one-third of them are unintended; out of them, 20% are terminated by induced abortion [5]. Consequently, around 47,000 women die each year from complications of unsafe abortions [6].

In developing countries, the World Health Organization (WHO) estimates that one woman dies every eight minutes due to unsafe abortions. Annually, around five million women who undergo unsafe abortions require hospitalization [7]. In these nations, ECP is the most commonly used method of EC after unprotected sexual intercourse to prevent unwanted and unintended pregnancy [4]. But still about one in six married women faces an unmet need for contraceptive [8].

Similarly, the Ethiopian demographic health survey 2016 (EDHS 2016) report showed that contraceptive prevalence rate among Ethiopian women aged 15–49 is 36% while the utilization of emergency contraceptive among sexually active unmarried women is 4% [9]. Even though contraceptive use among reproductive age group is increasing, induced abortion remains a commonly practiced fertility control method. Induced abortion is practiced at the rate of 45 per 1000 among women in the reproductive age group. This resulted in significant number of maternal morbidity and mortality [10].

Increasing the availability of emergency, contraceptive is important to reduce unwanted and unplanned pregnancy. Basically, it is the only option a woman has after unprotected sex [4]. Surprisingly, all women of reproductive health group, particularly young age women (10–24 years old), are affected by complication of unsafe abortion. Consequently, induced abortion can lead to various health problems such as chronic pelvic inflammatory diseases, mental disorders, subsequent adverse reproductive outcomes, and even secondary infertility [11]. As a measure, emergency contraceptive is one of the methods to prevent unplanned pregnancy when it is available and properly used. Because of the underutilization of emergency contraceptive, however, women are being exposed to unsafe abortion and its complications [12]. Most of the deaths that occur in developing countries are due to the lack of accessibility and availability of reproductive health services, especially family planning. Utilization of emergency contraceptive may play vital role in decreasing abortion and its complications [13].

Among the factors that are associated with the utilization of EC are urban dwellers, education, ever used contraception, and knowledge of contraception [8, 12, 14]. Age group greater than or equal to 25 years, married students, and students with unfavorable attitude were less likely users of EC [15]. Age, living arrangement, education, marital status, and religion are associated with the utilization of emergency contraceptive [5, 12]. Emergency contraceptive pills which are affordable, available, and culturally and religiously acceptable are associated with the use of EC pills [4]. However, little is known on this issue, particularly in Tigray region. Therefore, this study aims to assess the knowledge of and utilization of emergency contraceptive and its associated factors among women seeking induced abortion in public hospitals, Eastern Tigray, Ethiopia.

## 2. Materials and Methods

### 2.1. Study Setting, Period, and Population

Hospital-based cross-sectional study design was conducted from April to July 2017. Tigray region has total fertility rate of 4.7. Contraceptive prevalence rate of any method and ever usage of emergency contraceptive among currently married women in Tigray region is 36.3% and 0.00%, respectively [9]. The study was conducted in public hospitals found in eastern zone of Tigray. In this zone, there are two general hospitals, four primary hospitals, and health centres. All these health care settings provide safe termination of pregnancy. In this study, all those public hospitals in the zone were included. These are Adigrat general hospital, Wukro general hospital, Fasti primary hospital, Dowhan primary hospital, Hawzen primary hospital, and Mulu primary hospital. The source and study population were all women who came for safe termination of pregnancy in those public hospitals in the eastern zone of Tigray. All women who seek for safe termination of pregnancy were eligible for the study. Women who seek termination of pregnancy due to fetal abnormality, who were severely ill, and who were mentally ill were excluded from the study.

### 2.2. Sample Size Determination and Sampling Procedure

Sample size was calculated using single population proportion formula considering the following assumptions: 95% confidence interval, 5% of margin of error, and 34.1% prevalence of knowledge about emergency contraceptive among women coming for induced abortion in Dire Dawa [5]. Adding 10% of nonresponse rate, the final sample size was 380. The total sample size was allocated proportionally to each health facility based on their average monthly number of safe abortion performed in 2016/2017 G.C. Based on this, the sample size for Adigrat general hospital, Wukro general hospital, Hawzen primary hospital, Fatsi primary hospital, Mulu primary hospital, and Dowhan primary hospital was 172, 94, 24, 19, 38, and 33, respectively. A systematic sampling technique was used to get all study subjects in the abortion service unit of the hospitals.

### 2.3. Data Collection Procedure and Data Quality Control

A face-to-face interviewer-administered questionnaire was used to collect data from the study participants. This questionnaire was adapted from various related literatures [5, 16–20]. The questionnaire contains a total of 25 items with two parts. The first part of the questionnaire includes sociodemographic variables like age, ethnicity, residence, religion, marital status, educational level, occupation, monthly income, partner education, and occupation. The second part of the tool includes questions related to obstetric history, knowledge about EC, attitude towards EC, utilization of EC, and barriers for utilizing EC. Obstetrical data were extracted from the women's medical record. Primarily, this questionnaire was prepared in English. To assure its consistency, it was translated to Tigrinya and again translated back to English by different language experts. The data was collected by six diploma midwives and supervised by four MSc health professionals. Data collectors and supervisors had obtained training which was focused on the rational, objective, data collection instrument, and handling of ethical issue of the study. Pretest was also done on 5% of the total sample size in Adwa general hospital. Based on the result, modifications have been done to portion of the tool. Furthermore, data were checked for completeness and possible occurrence of mistakes at daily base.

### 2.4. Data Entry, Processing, and Analysis

The data were entered into Epi Info version 7 and exported to SPSS version 20 software packages for analysis. On bivariate analysis, variables showed that significant statistical association at *P* value less than 0.05 was entered to multiple logistic regression analyses to control the effect of confounding variables and identify variables independently associated with outcome variables. Statistically significant variables were reported using adjusted odds ratio with 95% of confidence interval and *P* value <0.05. Finally, results were presented using texts, charts, and tables. Mean and standard deviation were reported for normally distributed variables.

### 2.5. Operational Definition

Operational definitions are as follows: “knowledgeable to EC” refers to women who answered correctly and their scores are above or equal to the mean score of the total 8 knowledge questions; “not knowledgeable” refers to women who answered correctly and their scores are below the mean score of the total knowledge questions [5, 21].

Altitude about EC was measured using six items related on three-point Likert scale as agree, neutral, and disagree. Respondents were considered to have positive attitude if their scores are greater than or equal to the mean and negative attitude if their scores are below the mean [5, 21].

“Utilization of emergency contraceptive” refers to women who ever used emergency contraceptive after unprotected sexual intercourse to prevent unintended pregnancy in their life time.

## 3. Result

### 3.1. Sociodemographic Characteristics of the Respondents

Out of the total 380 women, 369 of the respondents completed the interview and the overall response rate was 97.1%. Among the nonrespondents, 5 participants are from Adigrat general hospitals, 3 are from Wukro general hospital, and the remaining are from Mulu primary hospital. The respondents' age ranged from 16 to 47 years old with mean and standard deviation of 25.2 ± 6.95. One hundred eighteen (32%) of the respondents were in the age group 20–24 while 198 (53%) are rural dwellers. The majority of the women, 294 (79.7%), were Tigraway in ethnicity. The marital status for more than half of the women, 217 (58%), is single while the majority of the participants, 302 (81.8%), were Orthodox-Christian religion followers. Regarding their educational status, 132 (35.8%) of the women achieved secondary school education (Table 1).

### 3.2. Obstetric History of Respondents and Reason for Current Termination of Pregnancy

Half of the respondents, 188 (50.9%), were primigravida. One hundred ninety-five (52.8%) of the study participants had no parity. Twenty-five (60.8%) of the respondents had a history of previous abortion. Out of those, 9 (76%) had spontaneous abortion. The reasons for the current termination of pregnancy were as follows: incest (126 (34.1%)), followed by rape (104 (28.1%)), economical problem (38 (10.3%)), having breast feeding child (33 (8.9%)), under age (16 (4.3%)), unplanned pregnancy (15 (4.1%)), decision with husband (9 (2.4%)), husband influence (9 (2.4%)), maternal mental and physical problem (2 (0.8%)), and others (17 (4.6%)).

### 3.3. Knowledge of Respondents about EC among Women Seeking Induced Abortion

Out of the total 369 respondents, only 126 (34.1%) heard about emergency contraceptive. The source of information for 71 (56.3%) of the respondents was health care workers. Regarding their knowledge about the dose of emergency contraceptive, 258 (69.9%) of them did not know how many doses are given, 20 (5.4%) answered one dose, 86 (23.3%) answered two doses, and the rest said three doses. Two hundred sixty-four (71.5%) of the respondents did not know the time interval between the first dose and second dose (Table 2).

**Figure 1:**
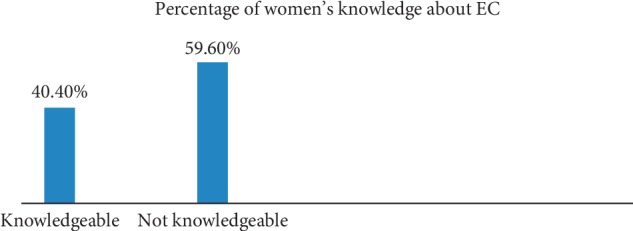
Respondents' knowledge about EC among women seeking abortion in public hospitals in eastern zone of Tigray regional state, Ethiopia, 2017 (*n* = 369).

One hundred twenty (32%) of the respondents had got health education about emergency contraceptive. The mean score of attitude towards emergency contraceptive was 0.68. Two hundred fifty-four (68.8%) of the respondents had positive attitude towards emergency contraceptive. The mean and standard deviation knowledge scores for the study participants were 0.4 and 0.491, respectively. One hundred forty-nine (40.4%) of the respondents were knowledgeable about emergency contraceptive with 95% of CI (0.35–0.45) (Figure 1).

### 3.4. Utilization of EC among Women Seeking Induced Abortion

The magnitude of emergency contraceptive utilization among women who came for induced abortion was 45 (12.2%). Among women who utilized EC, 43 (95.6%) had taken two pills, 44 (97.8%) had taken the pills twelve hours apart, and 1 (2.2%) had taken the pill after 24 hours from the first pill. In addition, thirty-two (71.1%) of the women had taken the pill within three days after unprotected sex while 12 (26.7%) had taken the pill within two days after unprotected sex and 1 (2.2%) had taken the pill before sex.

### 3.5. Factors Associated with Utilization of EC among Women Seeking Induced Abortion

Sociodemographic variables like place of residence, ethnicity, religion, educational level, respondent's occupation, partner education level, knowledge, and received health education about EC were significantly associated by using the bivariate regression analysis. But after controlling for the effects of potentially confounding variables using multivariate logistic regression, religion of the respondents, knowledge, and previous utilization contraceptive method were significantly associated with the utilization of emergency contraceptive. Protestant religion followers were 60.8 times more likely to utilize EC in comparison with Orthodox-Christian followers (AOR = 60.85, CI (5.34–693.29)). Women who did not use any contraceptive method previously were 87% less likely to use EC compared with counterparts (AOR = 0.13, CI (0.05–0.36)). Women who were not knowledgeable about EC were 97% less likely to utilize EC than those who were knowledgeable about EC (AOR = 0.030, CI (0.006–0.14)) (Table 3).

## 4. Discussion

Unintended pregnancy is the main challenge to reproductive health especially of youth in low-income countries particularly in sub-Saharan Africa. Some of women who had unintended pregnancy perform abortion. Many of them performed unsafe abortion and others may continue the pregnancy to term. Lastly, they face different complications like maternal morbidity and mortality [22, 23]. Unmet need for family planning among currently married women in Ethiopia and Tigray region is still 22.3 and 18%, respectively [9]. So, emergency contraceptive is the best option to prevent such a problem when taken within recommended dose and time interval after unprotected sexual intercourse. In this study, respondents' age was from 16 to 47 years old with mean and standard deviation of 25.2 ± 6.95. Fifty-three percent of the respondents were rural dwellers.

In this study, the magnitude of women who were knowledgeable about emergency contraceptive was 40.4% and 95% CI (35%, 45%). This is more or less similar to the study done in Dire Dawa (34.1%) [5]. But this is much higher than the study conducted in India (5.5%) [24]. This could be due to the fact that the cultural acceptance of family planning is higher in Ethiopia and there is a time gap between the two studies. In addition, it is higher than the study conducted in South Africa 15% and in Egypt 24.5% [1, 19]. This could be due to the combined effort of health care providers and health extension workers towards reproductive health issue in Ethiopia. Similarly, the finding of this study is also higher than the study conducted in Jimma university specialized hospital, southwest Ethiopia (10.1%) [22]. This might be due to the fact that our study population is taken from multiple health institutions and takes higher sample size. The finding of this study on the knowledge of women regarding emergency contraceptive is lower than that from Ghana (69%) [4]. This gap might be due to sociodemographic characteristics distribution difference between Ethiopia and Ghana.

The proportion of ever utilization of emergency contraceptive in this study is 12.2% and 95% CI (9%, 16%). The finding of this study is higher than the study conducted in India where none of them used emergency contraceptive [24]. This might be due to the fact that religious acceptance of family planning is higher in Ethiopia than in India. Other justification could be sociodemographic characteristics difference between Ethiopia and India. The finding of this study on ever utilization of emergency contraceptive is higher than the study conducted in South Africa (4%) [1]. The reason could be the difference in knowledge level of the women towards emergency contraceptive between the nations. However, it is similar with the study conducted in Ethiopian Dire Dawa and Immigration and Nationality Affairs Office (9.7 and 9.3%, respectively) [5, 8].

In this study, women who are not knowledgeable about emergency contraceptive were less likely to utilize EC. This finding is similar to the study done in Dire Dawa, Immigration and Nationality Affairs Office Ethiopia, India, and Sweden [5, 8, 14, 25]. Women who had not use contraceptive method previously were also less likely to use emergency contraceptive. This finding is in line with the study done at Mizan-Tepi University and South West Ethiopia [8, 26]. Protestant in religion was more likely to use EC pills in this study. It is in line with the study done in Ghana [4]. This could be justified by religious acceptance of the utilization of EC pills in protestants than other religions.

## 5. Conclusion

Emergency contraceptive has an advantage of preventing unintended pregnancy and prevents induced abortion after unprotected sexual intercourse. But the majority of the respondents were not knowledgeable about emergency contraceptive pills. The utilization of emergency contraceptive pill is very low among study participants. Women who are not knowledgeable and who did not utilize any contraceptive method previously were less likely to utilized emergency contraceptive pills. Protestant religion followers were likely to utilize emergency contraceptive.

## Figures and Tables

**Table 1:** Sociodemographic characteristics of the respondents among women seeking induced abortion in public hospitals in eastern zone of Tigray regional state, Ethiopia, 2017 (*n* = 369).

Variable	Frequency	Percent
Age (years)		
15–19	88	23.8
20–24	118	32
25–29	67	18.2
30–34k	41	11.1
35–39	38	10.3
≥40	17	4.6

Religion		
Orthodox Christian	302	81.8
Catholic	41	11.2
Muslim	19	5.1
Protestant	7	1.9

Marital status		
Single	217	58.8
Married	134	36.3
Divorced	17	4.6
Widowed	1	0.3

Educational level		
Unable to read and write	96	26
Able to read and write	39	10.6
Primary school (1–8 grade)	68	18.4
Secondary school (9-10 grade)	132	35.8
Diploma and above	34	9.2

Occupation		
Students	105	28.5
House wife	138	37.4
Governmental employed	31	8.4
Private employed	61	16.5
Others^*∗*^	34	9.2

Partner education (*n* = 134)		
Unable to read and write	44	32.8
Able to read and write	47	35.2
Primary (1–8 grade)	18	13.4
Secondary (9-10 grade)	16	11.9
Diploma and above	9	6.7

Partner occupation (*n* = 134)		
Farmer	66	49.3
Daily labor	37	27.6
Merchant	12	9
Governmental employed	11	8.2
Others^*∗*^	8	5.9

**Table 2:** Knowledge of respondents about EC among women seeking induced abortion in public hospitals in eastern zone of Tigray regional state, Ethiopia, 2017.

Variable	Frequency	Percent
Source of information		
Health workers	71	56.3
Radio/TV	52	41.3
Friends	44	34.9
Health extension workers	17	13.5
Family members	10	7.9
Neighbor	6	4.8
Husband	6	4.8

What do you do after unprotected sexual intercourse? (≥ one answer is possible)		
Terminate the pregnancy^*∗*^	134	36.3
I do nothing^*∗*^	75	20.3
Use EC pills^*∗*^	70	19
I don't know	85	23
Others	5	1.4

Advantage of EC		
Prevent pregnancy^*∗*^	174	47.2
Regular contraceptive	18	4.9
Prevent STIs	15	4.0
Terminates pregnancy	19	5.1
I don't know	143	38.8

What is the time limit to take EC?		
At any time	27	7.3
Before sex	10	2.7
Within 24 hours after sex	26	7
Within 72 hours after sex^*∗*^	75	20.3
Within 5 days after sex	15	4.1
I don't know	216	58.5

Time interval between first dose and second dose		
12 hours	79	21.4
24 hours	26	7.1
I don't know	264	71.5

When do we use EC? (≥ one answer is possible)		
When raped^*∗*^	116	31.4
When condom breaks^*∗*^	111	30.1
When we miss pills^*∗*^	22	6
When there is no contraceptive^*∗*^	38	10.3
I don't know	199	53.9

^*∗*^The correct possible answer.

**Table 3:** Factors associated with the utilization of EC among women seeking induced abortion in public hospitals in eastern zone of Tigray regional state, Ethiopia, 2017 (*n* = 369).

Variable	Utilization of EC		COR (95% CI)	AOR (95% CI)
	Yes	No		
Age				
15–19	7 (8%)	81 (92%)	1	
20–24	19 (16.1%)	99 (83.9%)	2.22 (0.88–5.54)	
25–29	11 (16.4%)	56 (83.6%)	2.27 (0.83–6.22)	
≥30	8 (8.3%)	88 (91.7%)	1.05 (0.36–3.30)	

Place of residence				
Urban	31 (18.1%)	140 (81.9%)	1	1
Rural	14 (7.1)	184 (92.9%)	0.34 (0.17–0.67)^*∗*^	0.67 (0.30–1.48)

Ethnicity				
Tigray	35 (11.9%)	259 (88.1%)	1	1
Erob	5 (7.9%)	58 (92.1%)	0.63 (0.24–1.69)	4.34 (0.73–25.88)
Amhara	4 (44.4)	5 (55.6%)	5.92 (1.51–23.09)^*∗*^	4.89 (0.12–199.22)
Afar	1 (33.3%)	2 (66.7%)	3.70 (0.32–41.87)	0.54 (0.080–3.74)

Religion				
Orthodox Christian	34 (11.3%)	268 (88.7%)	1	1
Catholic	3 (7.3%)	38 (92.7%)	0.62 (0.18–2.12)	0.63 (0.08–1.61)
Muslim	4 (21.1%)	15 (78.9%)	2.10 (0.65–6.70)	2.37 (0.42–13.39)
Protestant	4 (57.1%)	3 (42.9%)	10.5 (2.25–48.96)^*∗*^	60.85 (5.34–693.29)^*∗∗*^

Educational level				
Unable to read and write	1 (1%)	95 (99%)	0.02 (0.003–0.17)^*∗*^	0.21 (0.02–2.16)
Able to read and write	3 (7.7%)	36 (92.3%)	0.17 (0.04–0.69)^*∗*^	0.54 (0.10–2.78)
Primary (1–8 grades)	13 (19.1%)	55 (80.9%)	0.49 (0.19–1.26)	1.90 (0.57–6.29)
Secondary (9–10 grades)	17 (12.9%)	115 (87.1%)	0.30 (0.12–0.74)^*∗*^	0.56 (0.19–1.62)
Diploma and above	11 (32.4%)	23 (67.6%)	1	1

Marital status				
Single	32 (14.7%)	185 (85.3%)	1	
Married	11 (8.2%)	123 (91.8%)	0.517 (0.251–1.064)	
Divorced	2 (11.1%)	16 (88.9%)	0.723 (0.159–3.295)	

Occupation				
Students	11 (10.5%)	94 (89.5%)	1	1
House wife	12 (8.7%)	126 (91.3%)	0.81 (0.34–1.92)	1.04 (0.28–3.81)
Governmental employed	7 (22.6%)	24 (77.4%)	2.49 (0.87–7.11)	0.16 (0.02–1.13)
Private employed	9 (14.8%)	52 (85.2%)	1.47 (0.57–3.80)	0.38 (0.10–1.41)
Daily labor	3 (42.9%)	4 (57.1%)	6.40 (1.26–32.45)^*∗*^	1.37 (0.20–9.25)
Commercial sex workers	2 (14.3%)	12 (85.7%)	1.42 (0.28–7.21)	0.31 (0.03–2.91)
Others	1 (7.7%)	12 (92.7%)	0.71 (0.08–6.01)	1.54 (0.0.11–21.35)

Got health education about EC				
Yes	32 (26.7%)	88 (73.3%)	1	1
No	13 (5.2%)	236 (94.8%)	0.15 (0.07–0.30)^*∗*^	0.78 (0.30–2.05)

Previous utilization of any contraceptive				
Yes	38 (20.4%)	148 (79.6%)	1	1
No	7 (3.8%)	176 (96.2%)	0.15 (0.06–0.35)^*∗*^	0.13 (0.05–0.36)^*∗∗*^

Average knowledge				
Knowledgeable	42 (28.2%)	107 (71.8%)	1	1
Not knowledgeable	3 (1.4%)	217 (98.6%)	0.03 (0.01–0.11)^*∗*^	0.03 (0.006–0.14)^*∗∗*^

Attitude				
Positive	35 (13.8%)	219 (86.2%)	1	
Negative	10 (8.7%)	105 (91.3%)	1.67 (0.80–3.51)	

^*∗*^Significant at *P* value <0.05 on bivariate. ^*∗∗*^Significant at *P* value <0.05 on multivariate analysis. OR: odds ratio.

## Data Availability

The data used to support the findings of this study are available from the corresponding author upon request.
